# Extreme diversity in the songs of Spitsbergen's bowhead whales

**DOI:** 10.1098/rsbl.2018.0056

**Published:** 2018-04-04

**Authors:** K. M. Stafford, C. Lydersen, Ø. Wiig, K. M. Kovacs

**Affiliations:** 1 Applied Physics Laboratory, University of Washington, Seattle, WA 98105, USA; 2 Norwegian Polar Institute, NO-9296 Tromsø, Norway; 3 Natural History Museum, University of Oslo, N-0318 Oslo, Norway

**Keywords:** bowhead whale, Arctic, *Balaena mysticetus*, song

## Abstract

Almost all mammals communicate using sound, but few species produce complex songs. Two baleen whales sing complex songs that change annually, though only the humpback whale (*Megaptera novaeangliae*) has received much research attention. This study focuses on the other baleen whale singer, the bowhead whale (*Balaena mysticetus*). Members of the Spitsbergen bowhead whale population produced 184 different song types over a 3-year period, based on duty-cycled recordings from a site in Fram Strait in the northeast Atlantic. Distinct song types were recorded over short periods, lasting at most some months. This song diversity could be the result of population expansion, or immigration of animals from other populations that are no longer isolated from each other by heavy sea ice. However, this explanation does not account for the within season and annual shifting of song types. Other possible explanations for the extraordinary diversity in songs could be that it results either from weak selection pressure for interspecific identification or for maintenance of song characteristics or, alternatively, from strong pressure for novelty in a small population.

## 1. Background

Complex ‘song’ in mammals is rare. While many mammalian taxa produce repetitive ‘calls’, sometimes called advertisement songs [1–3], few mammals produce vocal displays akin to bird song, which is defined by multiple frequencies and amplitude-modulated elements combined into phrases and organized in long bouts [4]. Such songs have been documented in only a few mammalian species, including some bats (Chiroptera), gibbons (Hylobatidae), mice (*Scotinomys* spp.), rock hyraxes (*Procavia capensis*), and two great whales, humpback (*Megaptera novaeangliae*) and bowhead (*Balaena mysticetus*) whales [3,5–8]. With the exception of gibbons, in which males and females duet [5], complex songs in mammals are thought to be produced only by males [6,9–11]. Male mammals are thought to sing to defend territories, advertise their quality, attract mates or some combination of these functions [5,11].

The song in baleen whales has been studied extensively only in humpback whales, which sing similar songs within a season across a whole population. The structure of that song gradually evolves over the season in unison [12] and transfer of song types has been documented to occur directionally from one population to another over a period of years [13]. Humpback whale songs are composed of a hierarchy from units to sub-phrases to phrases to themes [12].

Less is known about the songs of bowhead whales compared with humpback whales, but bowhead whale songs generally consist of a single phrase that includes amplitude- and frequency-modulated elements repeated in bouts, with two different sounds often produced simultaneously [14]. A pilot study from the Fram Strait in 2008–2009 provided the first indication that tens of song types were produced by bowhead whales in this region within a single overwinter period [8]. No year-round studies of song diversity exist for other bowhead whale populations although multiple song types in a single year have been documented for two other populations [15,16]. Herein, we document extremely high inter- and intra-annual diversity in the mammalian song from the Spitsbergen bowhead whale population.

## 2. Material and methods

Omni-directional hydrophone recorders were deployed and redeployed annually from 2010 to 2014 in September on a long-term oceanographic mooring in western Fram Strait, at about 78°49 N, 5° W (electronic supplementary material, figure S1). The mooring deployed in 2011 was not recovered. Instruments recorded acoustic data for the first 14–17 min h^−1^ throughout the year. Upon recovery, the data were downloaded and spectrograms (10–4000 Hz, 2048 point FFT, 50% overlap, Hann window) were created for each data file. Spectrograms were then reviewed visually for the presence of bowhead whale songs that were classified by eye based on time-frequency characteristics of each song type [17]. Files with ‘loud’ songs (possessing distinct units, song bandwidth exceeding 500 Hz) were analysed further to determine the unit structure and song composition. Individual songs were assigned a unique number if more than one iteration occurred (e.g. Fram2012-11 was the 11th song recorded in the 2012–2013 season while Fram2012-54 was the 54th; electronic supplementary material, S1–S4). Each song type was characterized by frequency, duration and amplitude and/or frequency modulation, number of units and phrases and compared to all other song types within and between years to determine the minimum number of different song types detected and to describe the diversity of songs in this species at this location.

## 3. Results

**Figure 1.**
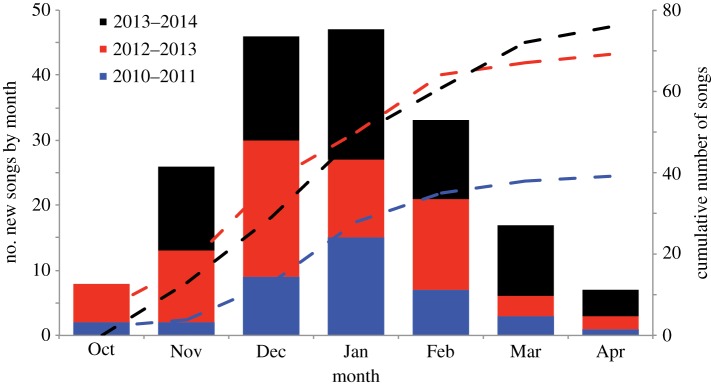
Total numbers of bowhead whale song types recorded in each month (bars) and cumulative number of song types (dashed lines) by year. The greatest number of different song types occurs in December and January, presumed to be the peak of mating season for bowhead whales.

Bowhead whale songs were detected 24 h per day throughout most of the winter every year (electronic supplementary material, figure S2). The greatest number of different song types was recorded in December and January (figure 1). A total of 184 different song types were recorded in the 3-year study period. Each song type was recorded in only one deployment period.

There was interannual variation in the number and timing of songs. The fewest songs were recorded in 2010–2011 (39 song types total, 895 recordings). Both 2012–2013 (69 song types total, 1338 recordings) and 2013–2014 (76 song types total, 998 recordings) had approximately twice as many different songs (figure 1).

**Figure 2.**
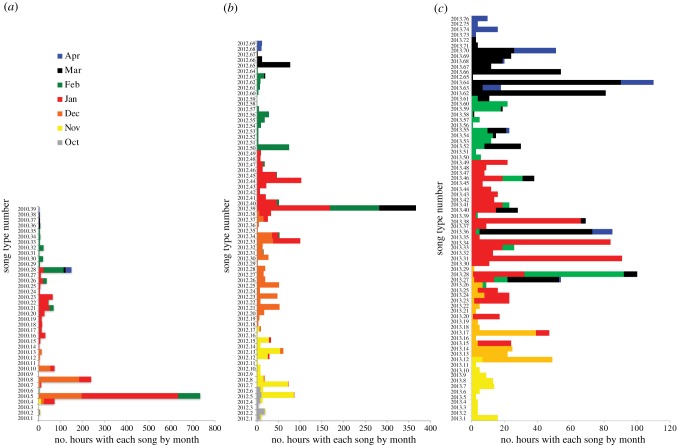
Total number of hours and months during which each song type was recorded by year. In most cases, a song type was only recorded in one month, though in some instances the same song type was recorded in two to four different months. (*a*) 2010–2011: 38 song types were recorded; (*b*) 2012–2013: 69 song types were recorded; (*c*) 2013–2014: 76 song types were recorded.

While most song types were short-lived—from hours to days—and seldom lasted longer than a month (figure 2), every year a few song types persisted throughout the winter. The overall trend for all years was a progression of song types appearing and then disappearing over time, with the greatest within-year diversity occurring in January for all 3 years examined.

Of the 3231 recordings containing songs over the 3 years, slightly over half (53%) contained only a single song type, while two different songs occurred in 37% of the recording periods. Less than 10% of all recordings contained more than two different song types.

## 4. Discussion

The diversity and interannual variability in songs of bowhead whales in this 3-year study are rivalled only by a few species of songbirds [4]. Among other mammalian singers, mice and gibbons tend to produce highly stereotyped and repetitive songs with few elements (e.g. [3,5]). Variation in rock hyrax and bat songs is primarily through changes in the arrangement of units [6,11]. Humpback whales produce complex songs that are similar within a year [7,12,13]. Although the repertoire of any one individual bowhead whale in this study cannot be determined, the catalogue of song types (184) is remarkably varied.

It is not known whether individual bowhead whales sing multiple song types in a season, but some are known to share the same song type in the same period in the Bering–Chukchi–Beaufort (BCB) population [14,16]. Nor is it known if individual bowhead whales maintain the same song throughout their lifetime or if they switch within and/or between years.

One explanation for the very high song diversity in the Spitsbergen bowhead whale population could be that the animals occupying this area in modern times include immigrants from both the BCB and the eastern Canada–western Greenland bowhead populations. Until recently, these populations have been assumed to be isolated from each other due to extensive, impenetrable sea ice cover in the High Arctic. However, in the past few decades, extreme declines in sea ice extent and thickness may have facilitated contact between these populations [18]. However, even if this region contains bowhead whales from multiple populations, this does not fully explain the high numbers of different song types recorded in this study or the lack of recurrence of song types from year to year.

It is plausible that the bowhead whales in the Fram Strait are simply a remnant of the original Spitsbergen population that survived the extreme historical levels of exploitation [19]. The influence of small population size on song diversity is conflicted; some studies suggest song diversity increases in smaller populations, although others have found that reduced or isolated populations exhibit a reduction in song diversity and produce simpler songs [20,21].

In some species, females appear to prefer a diverse song repertoire [22–25], suggesting that increased complexity of singing might confer reproductive advantages. A recent study of howler monkeys (*Alouatta* spp.) documented trade-offs in male reproductive characteristics based on (temporary) social structure: in groups with fewer males, or smaller social groups, males invested more in vocal displays as a reproductive tactic [26]. Normally, testes size and vocal repertoire (or other reproductive displays) are considered evolutionary trade-offs: depending upon social context, one of these may provide a selective advantage for individuals within a population over the other. For example, humpback whales have relatively small testes, and engage in physical competition as well as producing complex song displays, while right whales (*Eubalaena* spp.) have enormous testes, are drawn to ‘surface active groups' by a vocalizing female, and lack any apparent male acoustic display [27–29]. However, bowhead whales have both large testes and large vocal repertoires [30].

Bowhead whales are the only High Arctic resident baleen whale. Thus, interspecific identification via song may not confer the same selective advantage for bowheads that it might for other cetacean species. This could reduce selection pressure on song stereotypy, allowing for greater variation in song types as a result of a long-term cultural mutation in songs, or song novelty itself might confer an advantage [4,20,22,23].

Because bowhead whales sing underwater, in heavy ice during the polar night, a nuanced understanding of the variable syntax of this species will be difficult to obtain. Nevertheless, the singing behaviour of Spitsbergen bowhead whales, in which tens of distinct song types are produced annually, makes them remarkable among mammals.

## Supplementary Material

RSBL-2018-0056-supplementary figures for Extreme diversity in the songs of Spitsbergen's bowhead whales

## Supplementary Material

Sound file of song 2012-11 from Extreme diversity in the songs of Spitsbergen's bowhead whales

## Supplementary Material

Sound file of song 2012-25 from Extreme diversity in the songs of Spitsbergen's bowhead whales

## Supplementary Material

Sound file of song 2012-54 from Extreme diversity in the songs of Spitsbergen's bowhead whales

## Supplementary Material

Sound file of song 2012-57 from Extreme diversity in the songs of Spitsbergen's bowhead whales

## Data accessibility

Exemplar song files are deposited in Dryad Digital Repository (doi:10.5061/dryad.1ck400f) [31].
