# Supplementary material for: Extreme diversity in the songs of Spitsbergen's bowhead whales
Source: Biol Lett. 2018 Apr 4;14(4):20180056. doi: 10.1098/rsbl.2018.0056 (PMC5938564; doi:10.1098/rsbl.2018.0056)
Supplement: RSBL-2018-0056-supplementary figures for Extreme diversity in the songs of Spitsbergen's bowhead whales [file rsbl20180056supp1.docx]

Electronic Supplementary Material 1. Supplementary figures.

Extreme diversity in the songs of Spitsbergen’s bowhead whales

K.M. Stafford, C. Lydersen, Ø. Wiig, K. Kovacs


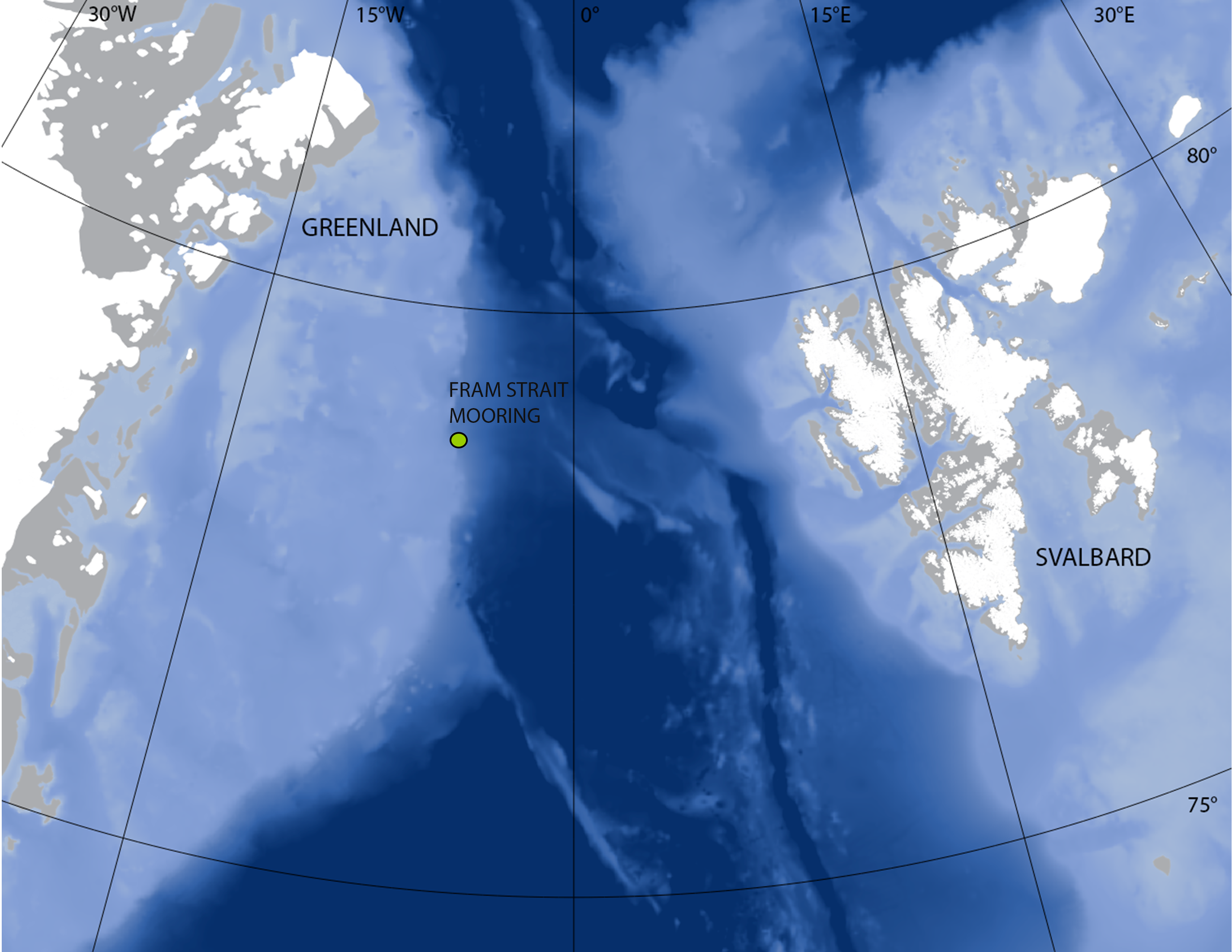


Figure S1. Map of Fram Strait study area with hydrophone location (green dot) for 2010-2014. This region is covered by close to 100% sea ice or at least it is within the marginal ice zone throughout the year and therefore is only accessible to ships in late summer. The hydrophone package was a Multi-electronique Aural-M2 (http://multi-electronique.com/aural.html) that was moored at a depth of approximately 80 m in water 1020 m deep. The instrument has a flat frequency response (+ 1 dB) from 5 Hz – 10,000 Hz and a sensitivity of -155 dB re 1 μPa.


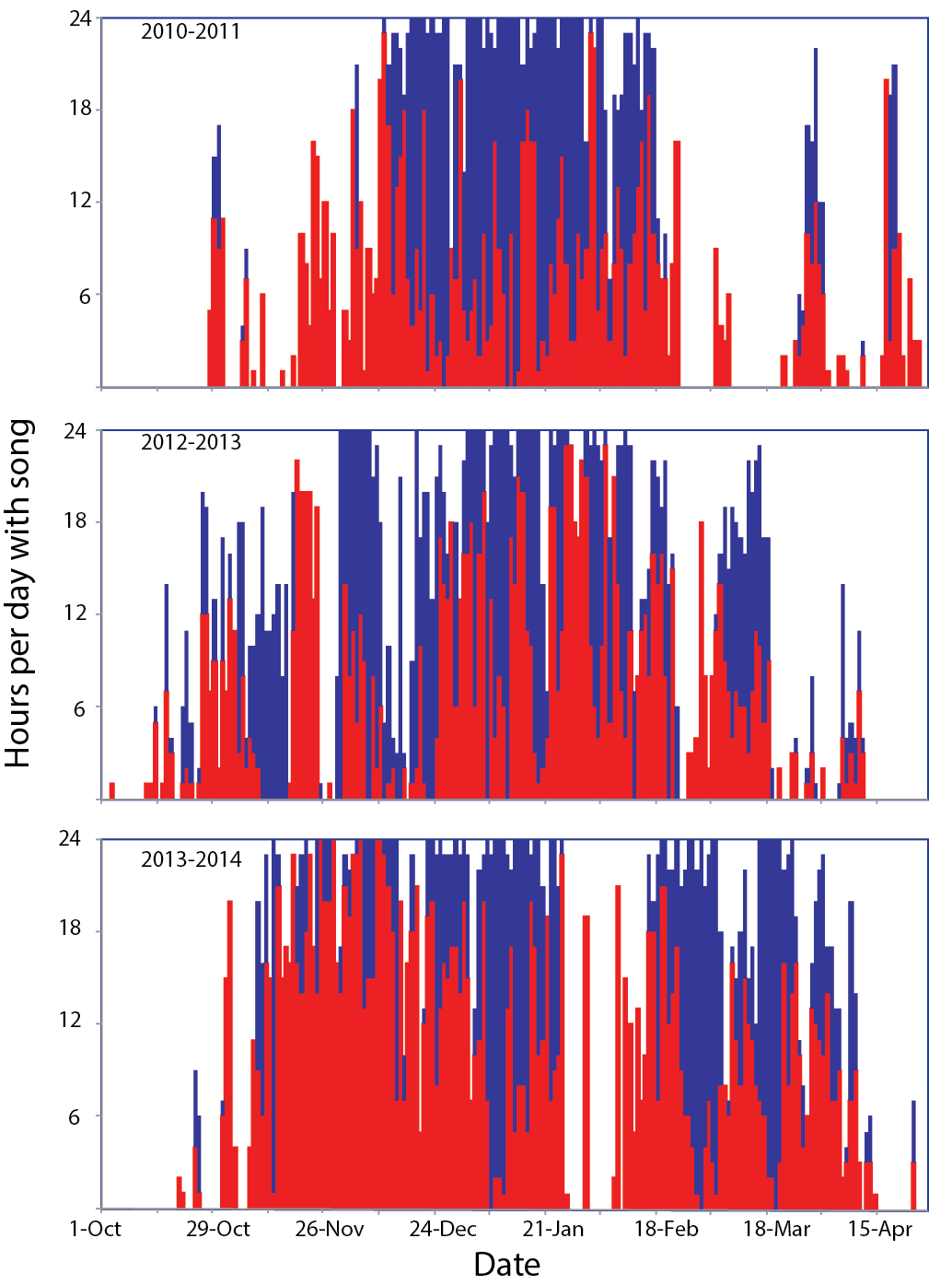


Figure S2. Number of hours per day with bowhead song from 1 October-30 April each year in Fram Strait. Data for 2012-2013 ended on 11 April. Red bars indicate hours in which loud songs were recorded, whereas blue bars represent the total number of hours (data files) per day in which singing was detected. For each day, there were 24 acoustic data files recorded with no data gaps, each lasting 14-17 min starting at the top of the hour. If a file contained song, the hour that file started was marked as “1”, if no song was recorded, then the file was marked as “0.” The number of hours (or files) per day with song and loud song were summed daily over the deployment period for each year.
